# An Interprofessional Case-Based Teaching Programme for the Multidisciplinary Team of an Acute Adult Inpatient Psychiatric Unit

**DOI:** 10.1192/bjo.2025.10258

**Published:** 2025-06-20

**Authors:** Oriana Bezzina, Venkatraghavan Ramaswamy

**Affiliations:** Tees, Esk and Wear Valleys NHS Foundation Trust, Durham, United Kingdom

## Abstract

**Aims:** An interprofessional educational intervention integrating case-based teaching was introduced to the multidisciplinary team (MDT) of two acute adult inpatient wards aiming to improve understanding, compassion and team dynamics through protected time for reflection, learning and social collaboration.

**Methods:** All MDT members were invited to a monthly 90-minute teaching session for a period of 7 months. Attendees could join in person or online. Sessions were primarily led by 2–3 MDT members from different professional backgrounds e.g. doctor, psychologist, and pharmacist. Two of the sessions included external presenters on specialist topics including eating disorders and substance misuse.

Sessions were split into: A Case Presentation on a current inpatient; A Topic Presentation on a subject linked to the case.

Feedback was collected anonymously on paper forms and emailed to online attendees following each session. Likert and free-text questions were used to gather feedback on teaching content, style, quality, relevance, and value. Following the final session, feedback was collected about the overall programme and presenters were asked to provide feedback on their experiences collaborating with colleagues. Likert items were analysed using Microsoft Excel and a basic thematic analysis was undertaken for free-text questions.

**Results:** On average, 13 professionals attended in-person and 3 online per session. Attendees included resident doctors, consultant psychiatrists, nursing staff, physical health nurse practitioners, psychologists, pharmacists, occupational therapists, support workers, activity coordinators and speech and language therapists. The average session ratings were 9.8/10 (1 – poor,10 –excellent) for usefulness and 9.75/10 for content, relevance, and teaching skill. Attendees who provided feedback on the overall programme (n=13) found it *extremely valuable* and wished for the sessions to continue. Five themes were identified: improved accessibility to teaching, shared perspectives, better understanding of professional roles, improved patient understanding via case-focussed teaching and reflection, and team building. Presenters (n=7) found the experience *very* or *extremely valuable* and they would recommend it to colleagues.

**Conclusion:** This interprofessional case-based teaching programme was successfully integrated within the acute inpatient setting. Effective interprofessional collaboration can be a key mechanism for improving service delivery, patient care and safety, and enhancing teams. Attendees found it beneficial discussing challenging/complex cases, improving their understanding of patients’ difficulties and colleagues’ roles. The sessions allowed attendees to reflect on cases with colleagues, sharing experiences and learning from one another positively impacting professional relationships. It would be useful for objective measures to be integrated within future research design to quantitatively measure changes in outcomes such as compassion, empathy, burn-out and teamwork.

